# The Lactococcal *dgkB* (*yecE*) and *dxsA* Genes for Lipid Metabolism Are Involved in the Resistance to Cell Envelope-Acting Antimicrobials

**DOI:** 10.3390/ijms22031014

**Published:** 2021-01-20

**Authors:** Aleksandra Tymoszewska, Tamara Aleksandrzak-Piekarczyk

**Affiliations:** Institute of Biochemistry and Biophysics, Polish Academy of Sciences (IBB PAS), Pawińskiego 5a, 02-106 Warsaw, Poland; tymoszewska@ibb.waw.pl

**Keywords:** aureocin A53- and enterocin L50-like bacteriocins, nisin, lactococcin 972, cell envelope-acting antibiotics, bacteriocins and antibiotic resistance, lipid metabolism, *Lactococcus lactis*

## Abstract

The emergence of antibiotic-resistant bacteria led to an urgent need for next-generation antimicrobial agents with novel mechanisms of action. The use of positively charged antimicrobial peptides that target cytoplasmic membrane is an especially promising strategy since essential functions and the conserved structure of the membrane hinder the development of bacterial resistance. Aureocin A53- and enterocin L50-like bacteriocins are highly cationic, membrane-targeting antimicrobial peptides that have potential as next-generation antibiotics. However, the mechanisms of resistance to these bacteriocins and cross-resistance against antibiotics must be examined before application to ensure their safe use. Here, in the model bacterium *Lactococcus lactis*, we studied the development of resistance to selected aureocin A53- and enterocin L50-like bacteriocins and its correlation with antibiotics. First, to generate spontaneous resistant mutants, *L.* *lactis* was exposed to bacteriocin BHT-B. Sequencing of their genomes revealed single nucleotide polymorphisms (SNPs) in the *dgkB* (*yecE*) and *dxsA* genes encoding diacylglycerol kinase and **1**-deoxy-D-xylulose **5**-phosphate synthase, respectively. Then, selected mutants underwent susceptibility tests with a wide array of bacteriocins and antibiotics. The highest alterations in the sensitivity of studied mutants were seen in the presence of cytoplasmic membrane targeting bacteriocins (K411, Ent7, EntL50, WelM, SalC, nisin) and antibiotics (daptomycin and gramicidin) as well as lipid II cycle-blocking bacteriocins (nisin and Lcn972) and antibiotics (bacitracin). Interestingly, decreased via the SNPs accumulation sensitivity to membrane-active bacteriocins and antibiotics resulted in the concurrently increased vulnerability to bacitracin, carbenicillin, or chlortetracycline. It is suspected that SNPs may result in alterations to the efficiency of the nascent enzymes rather than a total loss of their function as neither deletion nor overexpression of *dxsA* restored the phenotype observed in spontaneous mutants.

## 1. Introduction

The cell envelope is one of the most important cellular structures that gives a cell the shape, protects it from the environment, acts as a diffusion barrier and communication interface, and allows for cellular growth and division [1]. There are two groups of bacteria, Gram-positive and Gram-negative, that have fundamentally different cell envelopes. Both of them contain a cytoplasmic membrane composed of double layers of lipids such as phosphatidylglycerol, diphosphatidylglycerol (cardiolipin), and phosphatidylethanolamine, which are present in different proportions depending on the bacteria species [2]. The cell membrane of Gram-negative bacteria is surrounded by a cell wall composed of a thin layer of peptidoglycan and an outer membrane containing lipopolysaccharides. The Gram-positive bacteria do not contain a protective outer membrane, but their cell wall is much thicker. It is a multilayered, net-like structure composed of peptidoglycan and teichoic acids that can be either anchored to the cell membrane (lipoteichoic acids) or covalently bound to the peptidoglycan (wall teichoic acids) [1]. The bacterial cell envelope and its biosynthetic pathways are important targets for many antimicrobial agents such as antibiotics and bacteriocins, especially in Gram-positive bacteria that possess a cell envelope of two functional layers only.

Most of the conventional cell envelope targeting antibiotics inhibit different steps of cell wall biosynthesis. They can act on the intracellular targets such as fosfomycin that enters the cells through membrane channels/transporters and binds to MurA enzyme inhibiting the first step of peptidoglycan synthesis [3]. However, antibiotics that achieved the biggest clinical success act on the extracellular targets binding to important cell wall precursors or enzymes that process them [4]. The bacterial lipid II cycle constitutes a prime target for antibiotics. Ramnoplanin, vancomycin, and teicoplanin bind to the different moieties of lipid II [5,6], whereas bacitracin complexes with divalent metal ions, bind to undecaprenyl-pyrophosphate (UPP) and prevent its dephosphorylation to undecaprenyl phosphate (UP) [7]. β-lactams such as carbenicillin operate outside of the lipid II cycle by binding and inhibiting the activity of penicillin-binding proteins (PBPs), transpeptidases involved in the transpeptidation (cross-linking) step of the peptidoglycan synthesis [8]. Unfortunately, the long-term and improper use of antibiotics led to the emergence of antibiotics resistant bacteria, which is one of the biggest threats to global healthcare nowadays. Therefore, next-generation antibiotics with novel mechanisms of action are urgently needed. The cytoplasmic membrane is a particularly promising target for novel antibiotics. Its essentiality and highly conserved structure is a challenge for bacteria to modify it without substantial loss of function and, therefore, the acquisition of resistance is difficult [9]. A novel generation of membrane-targeting antibiotics includes daptomycin and gramicidin. Daptomycin is a front-line agent in the treatment of infections caused by methicillin-resistant *Staphylococcus aureus* (MRSA) and vancomycin-resistant *Enterococcus faecium* (VRE). Daptomycin complexes with calcium ions form micelles and then interact with anionic phosphatidylglycerol and disrupt membrane integrity [10]. Gramicidin forms dimeric channels in the membrane, facilitates diffusion of water and a selection of monovalent cations, and disrupts cellular ionic homeostasis [11].

In recent years, the use of antimicrobial peptides or proteins of bacterial origin, known as bacteriocins, as next-generation antibiotics has been intensively studied. Additionally, among bacteriocins, those targeting cell wall biosynthesis and cytoplasmic membrane can be distinguished. The most extensively studied cell envelope targeting bacteriocins are nisin and nisin-like lantibiotics. They are posttranslationally modified peptides with lanthionine and methyllanthionine residues forming tioether rings. They have a unique mode of action as they form a complex with a moiety of lipid II thereby inhibiting peptidoglycan synthesis and then incorporate into cytoplasmic membrane and form pores [12]. Lactococcin 972 (Lnc972) is the only known so far non-lantibiotic bacteriocin that targets lipid II and inhibits peptidoglycan synthesis but it does not form pores [13]. Amongst membrane-targeting bacteriocins, families of aureocin A53- (AurA53-) and enterocin L50- (EntL50-) like bacteriocins are currently getting attention because of their unique structures and biosynthetic mechanisms [14,15]. The first family consists of AurA53 [16], BHT-B [17], lacticin Q (LacQ) [18], lacticin Z (LacZ) [19], epidermicin NI01 (EpiNI01) [20], and lactolisterin BU (LliBU) [21]. The latter consists of EntL50 (EntL50A and EntL50B) [22], enterocin 7 (Ent7; Ent7A and Ent7B) [23], weissellicin M (WelM), and weissellicin Y (WelY) [24]. They are non-lantibiotic, leaderless bacteriocins with a highly cationic, hydrophobic character and broad spectra of antimicrobial activity. They display sequence homology within their families and all have similar saposin-like fold [15]. Saposins are a group of lipid-interacting human proteins with a structure of four-five α-helices forming a hydrophobic core (saposin fold) [25]. Saposin-like fold refers to a structure of three-four α-helices with a highly cationic, hydrophilic surface and a hydrophobic core that enables peptide–lipid interactions in the cell membrane [15,26,27]. Although all AurA53- and EntL50-like bacteriocins are thought to disrupt bacterial membranes in the absence of a specific receptor, the mechanism of membrane disruption by individual bacteriocins may differ. AurA53 acts through generalized membrane destruction rather than pore formation [28], while LacQ forms huge toroidal pores and causes accumulation of lethal hydroxyl radicals [29,30]. The mechanism of action of other AurA53- and EntL50-like bacteriocins remains to be elucidated.

To protect the essential and vulnerable structure of the cell envelope, bacteria have evolved complex regulatory networks that orchestrate cell envelope stress response. In Gram-positive *Firmicutes*, the BceRS- and LiaRS-like two-component regulatory systems (TCSs) are the core of cell envelope stress response. They mediate response to a specific compound or a group of related compounds (BceRS-like) as well as a wide array of cell envelope-damaging agents (LiaRS-like) [1]. They contain membrane-bound histidine kinases (HKs) and cytoplasmic response regulators (RRs). Typically, BceS- and LiaS-like HKs lack extracellular sensory domains and form sensory complexes with BceAB-like membrane-localized ABC transporters or LiaF-like membrane-anchored inhibitor proteins, respectively. The sensory complexes are responsible for sensing the stimuli and transferring a signal to the cognate BceR- and LiaR-like RRs that orchestrate the expression of genes responsible for maintaining cell integrity over a stressor presence. The genes encoding ABC transporters involved in the detoxification, proteins of the phage shock protein (Psp)-like response or proteins involved in the cell wall and cytoplasmic membrane synthesis and modification are among the most often regulated genes [31]. Modifications of the cell envelope composition are probably the most important mechanism of resistance since they often result in simultaneously decreased sensitivity to different antimicrobial agents including antibiotics and bacteriocins. One of the most important cell wall modifications is BceR-dependent D-alanylation of teichoic acids due to overexpression of the *dlt* operon. It decreases the negative charge of the cell wall making cells more resistant to positively charged antimicrobials. Additionally, LiaR-dependent increased peptidoglycan polymerization due to overexpression of the *pbp2* gene results in the cell wall thickening and increased antimicrobial resistance. The most important cytoplasmic membrane modification is a BceR-dependent lysination of the phosphatidylglycerol by the multiple peptide resistance factor (MprF) that results in the incorporation of the positive charge into phosphatidylglycerol [31]. Some less studied modifications of the cytoplasmic membrane include a lower amount of the saturated fatty acid and less elongated fatty acids due to lower expression of the *fab* operon [32], increased level of phosphatidylglycerol over cardiolipin [33], decreased level of the anionic phospholipids (phosphatidylglycerol and cardiolipin), and increased level of zwitterionic phosphatidylethanolamine [34,35]. The role of BceRS- and LiaRS-like TCSs in resistance to cell envelope targeting antibiotics is fairly well documented in many reviews on this topic [1,31,36]. However, among cell envelope-acting bacteriocins only nisin and Lcn972 were shown to induce stress response regulatory systems [32,37]. The mechanisms of resistance to other cell envelope targeting bacteriocins and the possible cross-resistance with antibiotics are not known. Understanding the biochemical and genetic basis of resistance to membrane-targeting AurA53- and EntL50-like bacteriocins is of great importance to allow the safe application of these bacteriocins as next-generation antibiotics.

Here we studied the genetic basis of resistance to the AurA53- and EntL50-like bacteriocins and a broad array of antibiotics with distinct mechanisms of action. First, we generated spontaneous resistant mutants by exposing highly sensitive *Lactococcus lactis* LMGT 3419 to BHT-B. By the use of genome sequencing, we identified responsible for the resistant phenotype non-synonymous single mutations within the *dgkB* (*yecE*) and *dxsA* genes involved in the lipid metabolism. Then, we tested the susceptibility of selected mutants to other AurA53- and EntL50-like bacteriocins including three functional (Ent7, EntL50, and WeiM) and two putative (K411 and SalC) representatives, nisin, Lcn972 and a wide array of antibiotics. This revealed that common mechanisms involving modification of the cell wall and cytoplasmic membrane may be involved in the resistance to tested bacteriocins and certain antibiotics, mainly those cell envelope-active. Moreover, we found here that gaining resistance to selected antimicrobials may increase sensitivity to some antibiotics, such as bacitracin, carbenicillin, and chlortetracycline.

## 2. Results

### 2.1. Accumulation of L. lactis BHT-B Resistant Mutants

Strains sensitive to BHT-B belong to the species *Micrococcus luteus, Lactococcus lactis, Streptococcus pyogenes,* and *S. equisimilis* [17]. *L. lactis*, formerly grouped into the genus *Streptococcus* [38], is a close relative to the BHT-B producing *Streptococcus rattus* BHT, and also is a model bacterium with a well-developed research tool-box dedicated to genetic analyses, therefore, was selected here to generate spontaneous mutants resistant to BHT-B. By growing the cells in the presence of bacteriocin in the solid medium, we isolated five independent *L. lactis* mutants (MUT70, MUT71, MUT72, MUT73, MUT78) with 4-fold decreased sensitivity to BHT-B in comparison with the parental strain *L. lactis* LMGT 3419 (minimum inhibitory concentration MIC_50_ = 6.3 μg/mL) (Table 1). Amidst these resistant mutants, *L. lactis* MUT78 was used for further mutagenization and, as the result, the second-generation *L. lactis* MUT78.2 mutant was obtained and characterized to have 2-fold and 8-fold decreased sensitivity in comparison with the parental *L. lactis* MUT78 and wild-type *L. lactis* LMGT 3419, respectively (Table 1).

### 2.2. Identification of Genes Altered by Mutations in the BHT-B Resistant Mutants

To identify genetic changes responsible for the development of resistance to BHT-B, genomes of all six spontaneous resistant mutants, as well as wild-type were isolated and sequenced. Subsequently, the whole-genome sequence reads from the resistant mutants were compared with the assembled reference genome to identify single nucleotide polymorphisms (SNPs). Mutations with low frequency or localized in low-coverage regions were excluded from further analysis. Two resistant mutants (MUT70 and MUT72) contained missense or nonsense mutations in the *yecE* gene encoding lipid kinase that resulted in the Asp96Tyr substitution or very early termination of translation after Thr54 of the YecE protein, respectively (Table 1). Three other mutants (MUT71, MUT73, MUT78) harbored a single and identical nonsense mutation in the *dxsA* gene encoding **1**-deoxy-D-xylulose **5**-phosphate (DXP) synthase (DXS). The effect of the mutation was a premature truncation of the DXP synthase at Ser490 position (Table 1). The second-generation MUT78.2 mutant was of special interest due to two, additional to *dxsA*, nonsense mutations in the *ysaB* and *ynhD* genes. Mutations resulted in the premature truncation of the encoded ATP-binding cassette (ABC) transporter permease YsaB and hypothetical protein YnhD (Table 1). More precise homology searches showed that YnhD shares 99.5% and 94.7% identity to, respectively, lactococcal 5-bromo-4-chloroindolyl phosphate hydrolysis protein (NCBI RefSeq WP_081042512.1) and XpaC-like protein (WP_011835023.1).

### 2.3. Analysis of Mutations in the yecE and dxsA Genes

**Figure 1:**
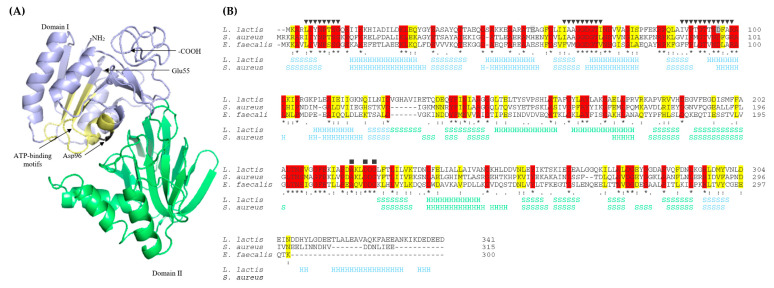
(**A**) The predicted tertiary structure of *L. lactis* LMGT 3419 DgkB (YecE). (**B**) Alignment of primary and secondary structures of diacylglycerol kinases from *L. lactis* LMGT 3419, *Staphylococcus aureus* MRSA252 and *Enterococcus faecalis* V583. Stars, colons, and dots indicate fully, high, and low consensus residues, respectively. Conserved and homologous amino acids are highlighted in red and yellow, respectively. Amino acids changed by nonsense and missense mutations are highlighted in light and dark grey, respectively. Triangles indicate three conserved motifs that participate in the nucleotide-binding in *S. aureus* MRSA252. Squares indicate a divalent cation binding site in *S. aureus* MRSA252. Secondary structure elements are indicated with H (α-helix) and S (β-strand). Structural domains I and II are distinguished with blue and light green, respectively.

To analyze the loci and therefore potential downstream effects of the mutations in the *yecE* and *dxsA* genes on the structure and function of the encoded protein, we predicted their structures using structural templates from the Protein Data Bank archive. YecE shares 45.54% and 33% identity with the used templates of the highest significance DgkB diacylglycerol kinases from *S. aureus* MRSA252 and *Enterococcus faecalis* V583, respectively. Due to this homology, from this point of this work, YecE will be named DgkB and accordingly, its encoding gene, *dgkB*. The tertiary structure of DgkB is of two-domain architecture with α/β-fold and an active site in a cleft between the two domains (Figure 1A). Based on the sequences and structures homology, we predicted the localization of three conserved nucleotide-binding motifs that are present in the N-terminal diacylglycerol kinase catalytic domain of DgkB, and define members of the soluble diacylglycerol kinase superfamily (Pfam accession no. PF00781) [39]. Importantly, both amino acids changed by mutations were localized in the N-terminal catalytic domain and, the substituted by missense mutation Asp96 (equivalent to Asp97 in *S. aureus*), was a part of the third conserved motif forming DgkB active site (Figure 1B).

**Figure 2:**
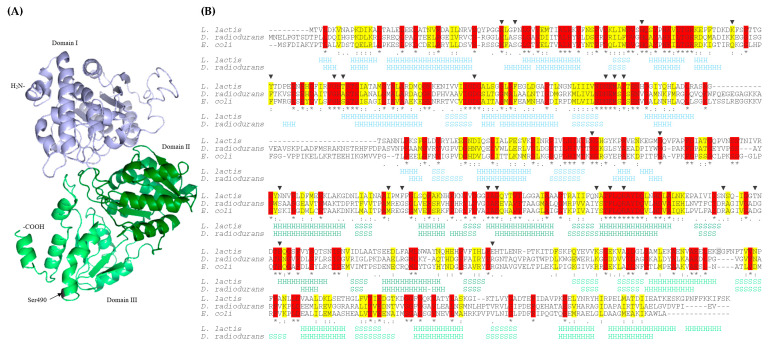
(**A**) Predicted tertiary structure of *L. lactis* LMGT 3419 DxsA. (**B**) Alignment of primary and secondary structures of DXP synthases from *L. lactis* LMGT 3419, *D. radiodurans* R1 and *E. coli* K12. Stars, colons, and dots indicate fully, high, and low consensus residues, respectively. Conserved and homologous amino acids are highlighted in red and yellow, respectively. Amino acid changed by nonsense mutation is highlighted in grey. Triangles indicate residues in the active site of *D. radiodurans* R1 and *E. coli* K12 DXP synthases. The secondary structure elements are indicated with H (α-helix) and S (β-strand). Structural domains I, II, and III are distinguished with blue, dark green, and light green, respectively.

DxsA shares 34.25% and 33.78% identity with used as templates of the highest significance DXP synthases from *Deinococcus radiodurans* R1 and *Escherichia coli* K12, respectively. DxsA contains three domains with central, mostly parallel β-sheets (five in I and III domain, and six in II domain) surrounded by α-helices (Figure 2A) and conserved amino acids forming DxsA active site [40]. Amino acid changed by nonsense mutation was not conserved among DXP synthases and was not localized nearby conserved amino acid residues forming an active site. While the active site was localized at the interface between the I and II domains, the mutated amino acid was localized at the beginning of the III domain of DxsA (Figure 2B). Therefore, nonsense mutation resulted in the production of truncated protein containing two domains only. Amino acid substitution in the DgkB active site, as well as the production of DxsA devoid of its C-terminal domain, could lead to significant changes in the activity of these proteins including their inactivation or induction of their activity.

### 2.4. Effect of Mutations on the Sensitivity to Bacteriocins

To examine an effect of mutations on the sensitivity to different cell envelope targeting bacteriocins, we tested the activity of membrane-disrupting AurA53- and EntL50-like bacteriocins (K411, Ent7, EntL50, WelM, and SalC), inhibiting cell wall synthesis Lcn972 and nisin against four representatives of *L. lactis* mutants (MUT70, MUT72, MUT78, MUT78.2). In comparison with the wild-type *L. lactis* LMGT 3419, all BHT-B-resistant mutants showed also decreased sensitivity to other bacteriocins tested. The level of sensitivity differed depending on the mutated gene. Mutations within *dgkB* (strains MUT70 and MUT72) had the lowest impact on the sensitivity to bacteriocins since only a 2–4-fold increase in MIC_50_ values was observed in most cases (Table 2). Mutation in *dxsA* (MUT78) resulted in the 4–8-fold MIC_50_ increase to all bacteriocins apart from EntL50 and nisin, whose presence elevated this ratio to over 32 (Table 2). Multiple mutations in MUT78.2 led to the highest increase of MIC_50_ values such as 8–16-fold to most bacteriocins except for Ent7, EntL50 and nisin in presence of which this ratio elevated to 32 (Ent7 and nisin) or 128 (EntL50) (Table 2).

### 2.5. Effect of Mutations on the Sensitivity to Antibiotics

To examine the effect of mutations on the sensitivity to antibiotics, *L. lactis* MUT70, MUT72, MUT78, MUT78.2 underwent susceptibility tests with a wide array of antibiotics acting on different cellular targets. We included the membrane-disrupting ones (daptomycin and gramicidin), inhibiting cell wall synthesis (amoxicillin, ampicillin, bacitracin, carbenicillin, cefuroxime, cephalothin, fosfomycin, and vancomycin), inhibiting DNA synthesis (ciprofloxacin and norfloxacin), inhibiting protein synthesis (chloramphenicol, chlortetracycline, clindamycin, erythromycin, gentamicin, kanamycin, streptomycin, and tetracycline), inhibiting folate synthesis (trimethoprim), and inhibiting respiration and pyruvate metabolism (nitrofurantoin). In comparison to the wild-type strain, all tested mutants exhibited increased sensitivity to bacitracin, carbenicillin, or chlortetracycline. The level of gained sensitivity depended on the mutated gene and, in the case of bacitracin, was most pronounced after mutation of *dgkB* (MUT72) or *dxsA*^-^*ysaB*^-^*ynhD*^-^ (MUT78.2), reaching 4–6-fold change of MICs (Table 2). In the presence of carbenicillin or chlortetracycline the most significant increase of sensitivity was triggered by multiple mutations within MUT78.2 and also by a single mutation in *dxsA* (MUT78). Contrary, in the presence of daptomycin, gramicidin, gentamicin, kanamycin, streptomycin, vancomycin, or fosfomycin, MIC values increased, indicating induction of mutants’ resistance to these antimicrobials. In this case, the most significant changes in the MIC values (>8–≥32-fold) occurred in the presence of daptomycin or gramicidin and considered mutants MUT78 and MUT78.2, both harboring mutated *dxsA*. Cross-resistance of mutants to gentamicin, kanamycin, streptomycin, vancomycin, or fosfomycin was on the lower level and reached mostly 2–4-fold of decreased sensitivity (Table 2). Finally, no significant changes in MICs of all remaining antibiotics tested were observed between the wild-type and mutants.

### 2.6. The Effect of Deletion or Overexpression of the dxsA Gene

The SNP occurring in *dxsA* reduced the susceptibility of the MUT78 strain to cell envelope-acting antimicrobials indicating its important role in the response to the presence of these compounds. However, this point mutation did not remove the DxsA protein completely but merely truncated its C-terminal end (Figure 2), which did not necessarily result in its inactivation. To examine the effect of bacteriocins and antibiotics on cells fully devoid of *dxsA*, first, we deleted it from the genome of *L. lactis* LMGT 3419. In comparison with the wild-type strain, the obtained deletion mutant *L. lactis* MUT80 showed no differences in the sensitivity to AurA53- and EntL50-like bacteriocins, nisin and Lcn972 (Table 2). A minor, at the most 2-fold increase of *L. lactis* MUT80 sensitivity to gramicidin, kanamycin, and streptomycin and 3-fold decrease of sensitivity to fosfomycin were observed (Table 2).

To examine the effect of bacteriocins and antibiotics on cells overexpressing the *dxsA* gene, we cloned it in the pIBB-JZK vector under the control of a strong cellobiose-responsive promoter P*ptcB* (pIBB-JZK:P*ptcB*:*dxsA*) in *L. lactis* LMGT 3419. In comparison to the wild-type *L. lactis* LMGT 3419 and MUT82 carrying an empty plasmid, obtained this way *L. lactis* MUT83 showed no differences in the sensitivity to all tested compounds (data not shown). On the other hand, the same recombinant construct pIBB-JZK:P*ptcB*:*dxsA*, complemented partially the point mutation of *dxsA* present in MUT78. Obtained in this way *L. lactis* MUT85 had a reduced level of resistance to almost all bacteriocins when compared to the strain carrying the empty pIBB-JZK (*L. lactis* MUT84) as well as parental *L. lactis* MUT78. However, the MIC_50_ values of *L. lactis* MUT85 did not fully recover to those of the wild-type strain, which indicates only a partial reversion to the wild-type phenotype. Moreover, this effect was observed only in the case of bacteriocins, whereas the level of *L. lactis* MUT85 resistance to antibiotics remained mostly unchanged when compared to *dxsA*^-^ strains (MUT78 and MUT84) (Table 2).

## 3. Discussion

Nowadays, an increasing number of infections caused by multidrug-resistant bacteria are reported. To treat them, next-generation antibiotics with novel mechanisms of action are needed. Positively charged antimicrobial peptides have become a promising group of antimicrobials due to their ability to kill bacteria via nonreceptor mediated disruption of the negatively charged cytoplasmic membrane [9]. Although the development of resistance to such peptides is difficult and complex, it is also inevitable. Therefore, in-depth studies are needed to understand and limit this phenomenon. Here, we studied the genetic basis of resistance to cationic, membrane targeting AurA53- and EntL50-like bacteriocins. Among them, for this study, we selected four functional (BHT-B, Ent7, EntL50, WelM) and two putative ones (K411 and SalC; UniProt accession numbers Q576C5 and A0A089RZU, respectively). *L. lactis* is a Gram-positive cocci and, due to its relatedness to some pathogens, sensitivity to many antibacterial compounds, as well as well-developed research tool-box dedicated to genetic analyses, this species is a model microorganism for research on resistance mechanisms. Moreover, since *L. lactis* is standardly used in food-processing industry, it may also acquire resistance to antimicrobial compounds added to food or produced by the bacteria present in these products. By growing sensitive *L. lactis* in the presence of BHT-B, we generated spontaneous resistant mutants, which revealed also cross-resistance to all other studied here bacteriocins as well as most of the cell-envelope targeting antibiotics. Interestingly, this time we were able to obtain mostly 2–32-fold more resistant mutants, whereas, in our previous research on the mechanisms of resistance to bacteriocins known as garvicins, we generated over 1024-fold more resistant mutants [41,42]. This difference may be due to the different targets of attack and mechanisms of action for these two groups of bacteriocins. While the receptor for garvicins is a membrane-located protein complex (mannose–phosphotransferase system; Man-PTS) [41,42], the AurA53- and EntL50-like bacteriocins do not have a specific receptor and act by inserting in and destroying the cell membrane [9,28,29,30]. Consequently, the mechanisms of resistance to these two groups of bacteriocins also differ. Whereas resistance to garvicins is achieved by simply inactivating or modifying the protein target of their attack [41,42], we speculate here that the mechanism for the AurA53- and EntL50-like bacteriocins may be the modification of the cell membrane lipid composition and/or cell wall structure. Specifically, we found that resistance to AurA53- and EntL50-like bacteriocins is due to mutations in the *dgkB* and *dxsA* genes. DgkB shares significant identity with soluble diacylglycerol kinases from *S. aureus* MRSA252 and *E. faecalis* V583 and owns conserved nucleotide-binding motifs that define members of the soluble diacylglycerol kinase superfamily. DgkB is a key enzyme in lipid metabolism that catalyzes the ATP-dependent phosphorylation of diacylglycerol (DAG) to phosphatidic acid. In bacteria, a large amount of DAG is formed by the hydrolysis of membrane phosphatidylglycerol in the process of lipoteichoic acids biosynthesis. Therein, phosphatidylglycerol is extensively used as the source of *sn*-1-glycerol-P headgroup. The synthesis of a single lipoteichoic acid requires the addition of 14 to 33 *sn*-1-glycerol-P headgroups and the removal of the headgroup from phosphatidylglycerol results in the formation of DAG. DgkB recycles DAG into the phospholipids biosynthetic pathway and thus prevents its accumulation in the bacterial cell membranes [39]. Although DgkB is an essential protein in bacteria, Jerga et al. [43] performed conditional inactivation of *dgkB* in *B. subtilis* and showed that it leads to the cessation of lipoteichoic acid formation, accumulation of DAG and eventual loss of cells viability. Our analysis showed that changed by missense mutation Asp96 (equivalent to Asp97 in *S. aureus*) may be a key residue of the lactococcal DgkB active site since in *S. aureus*, which possesses prototypical diacylglycerol kinase of Gram-positive bacteria, Asp97 coordinates Mg^2+^ via water molecules and is involved in the ATP binding forming the top of the nucleotide-binding site [39]. Identified in this study substitution of negatively charged, polar Asp with aromatic, partially hydrophobic Tyr may have a significant impact on the structure and function of the DgkB. In this study, we did not delete *dgkB*, as inactivation of DkgB is lethal due to accumulation of DAG or absence of lipoteichoic acids [43], and accordingly, it can be assumed that SNPs that arose in lactococcal genome did not abolish but rather modified the activity of this enzyme.

Our thorough analysis of the scientific literature shows a lack of data linking the *dgkB* gene with bacteriocin resistance to date. However, mutations within the gene encoding DgkB of *S. mutans* were previously identified in the spontaneously arising mutants with reduced susceptibility to chlorhexidine, a cationic antiseptic that targets bacterial cell membrane [44]. Similar to our study, almost all of these mutants displayed cross-resistance to daptomycin and increased sensitivity to bacitracin. Interestingly, spontaneous mutations occurred over the length of the *dgkB* gene and always resulted in amino acid substitutions, whereas one of the two SNPs found in this study resulted in a premature stop codon. The authors speculated that the mutations influenced the efficiency of the enzyme rather than caused its inactivation since although *dgkB* is an essential gene in *S. mutans* [45], no growth defects were observed in the *S. mutans* mutants expressing mutated *dgkB* [44]. This is also in line with our research hypothesis since SNPs that occurred in *dgkB* of *L*. *lactis* maintained the respective mutants viable. *S*. *mutans dgkB* mutants exhibited distinct amounts of lipoteichoic acid produced [44] and thus, it is tempting to speculate that SNPs in *dgkB* of *L. lactis* may also alter their accumulation in the cell wall. The modified amount of negatively charged lipoteichoic acids may be responsible for the changes in accessibility to the target molecules i.e., cytoplasmic membrane or lipid II cycle intermediates and/or influence the charge of the cell wall. The reduction of the negative charge of the cell wall could make cells more resistant to positively charged antimicrobials such as AurA53- and EntL50-like bacteriocins, gramicidin, nisin, Lcn972, and vancomycin. This mechanism seems more likely as it explains why spontaneous mutants exhibit also decreased sensitivity to positively charged aminoglycosides [46,47] such as streptomycin, kanamycin, and gentamicin and increased sensitivity to negatively charged carbenicillin and chlortetracycline. Nevertheless, further studies are needed to fully understand the mechanisms of DgkB-mediated resistance.

DxsA is a crucial enzyme in the isoprenoids biosynthesis. Isoprenoids are one of the larger groups of natural compounds that occur in all domains of life and take part in many important physiological processes. Well-known examples of bacterial isoprenoids include menaquinones (vitamin K2) and ubiquinones (coenzyme Q) that are involved in the production of energy through the electron transport chain [48], bactoprenols that act as membrane lipid anchors in the biosynthesis of peptidoglycan [49], and hopanoids that function as analogs of cholesterol in bacterial membranes [50]. All isoprenoids are synthesized from two precursors—isopentenyl pyrophosphate (IPP) and dimethylallyl pyrophosphate (DMAPP). DxsA catalyzes the first and the rate-limiting step in the non-mevalonate pathway for the biosynthesis of IPP and DMAPP that is the formation of **1**-deoxy-D-xylulose **5**-phosphate (DXP) from D-glyceraldehyde 3-phosphate and pyruvate [40,51,52]. Identified here, a nonsense mutation within the *dxsA* gene resulted in the production of a truncated protein with the preserved active site. As neither *dxsA* deletion nor overexpression recreated the changes in susceptibility to any of the antimicrobials that we observed in the spontaneous mutant, we assume that the DxsA enzyme in MUT78 is still functional. SNP that occurred in *dxsA* may result in the specific modification of the nascent protein function leading to the changes in the phenotype of the mutant. The effect of the modified DxsA on the change of mutant resistance persisted even in the presence of its wild copy in MUT85, which additionally confirms that truncated DxsA is active and efficiently performs its protective function. Due to the large number and diversity of bacterial isoprenoids, it is difficult to judge how a mutation in *dxsA* may be responsible for changes in sensitivity to studied bacteriocins and antibiotics. Anyway, the highest decrease in sensitivity was observed in the presence of membrane-active antimicrobials (AurA53- and EnL50-like bacteriocins, nisin, daptomycin, and gramicidin) therefore, modifications of the cell membrane seem to be the most likely mechanism of resistance. Hopanoid lipids are some of the most ubiquitous isoprenoids responsible for the cell membrane fluidity and permeability, thereby cell sensitivity to antibiotics and other stress conditions [50]. They can intercalate into a lipid bilayer, order saturated lipids and form a liquid-ordered phase in bacterial membranes. The presence of hopanoid lipids condenses, thickens, and decreases the fluidity and permeability of bacterial membranes [53]. Hopanoid-deficient mutants have been shown to display increased sensitivity to antibiotics such as polymyxin B, colistin (polymyxin E), erythromycin or chloramphenicol, detergents, and stress conditions [50,54,55,56]. Mutation within *dxsA* may boost hopanoids synthesis thereby membrane stability and integrity to counteract the disrupting activity of membrane-active antimicrobials.

In comparison to mutants carrying single mutations, *L. lactis* MUT78.2 with multiple SNPs had the highest level of changed susceptibility to studied antimicrobials suggesting that resistance mechanisms may accumulate. In *L. lactis* MUT78.2, in addition to a single SNP in *dxsA*, two additional mutations in the *ysaB* and *ynhD* genes were identified. Encoded by *ysaB*, an ABC transporter permease YsaB was previously shown to be part of the Bce-like stress response regulatory system that is involved in resistance to nisin and Lcn972. In *L. lactis*, it is composed of ABC transporter YsaCB and two-component regulatory system KinG-LlrG, while the latter consists of HK KinG and RR LlrG [32,37]. The primary role of this detoxification module is to mediate resistance to peptide antibiotic bacitracin. The BceAB transporter, which is a *B. subtilis* YsaCB homolog, binds to the bacitracin-UPP complex and releases UPP from the grip of the bacitracin, protecting the cell wall synthesis. At the same time, BceAB forms a sensory complex with BceS HK that uses a flux-sensing mechanism to monitor the detoxification capacity of BceAB and, if necessary, phosphorylates BceR to activate expression of the *bceAB* genes [57,58]. In accordance, truncation of lactococcal YsaB permease in MUT78.2 significantly increased mutant’s sensitivity to bacitracin. Point mutations in the *ysaCB-kinG-llrG* were speculated to activate expression of LlrG-regulated genes that in turn confer resistance to Lcn972 [59]. We anticipate that a similar mechanism may be involved in resistance to AurA53- and EntL50-like bacteriocins. BceR of *B. subtilis* regulates only the expression of *bceAB* genes, however, many BceRS-like TCSs regulate the expression of other genes such as *mprF* and *dltABCDE* [31]. As these genes lower the negative cell surface charge by modifying the cell wall and cytoplasmic membrane components [31], it is tempting to speculate that they are also regulated by the lactococcal KinG-LlrG TCS, which would explain decreased sensitivity of MUT78.2 to positively charged bacteriocins, daptomycin-calcium complexes, vancomycin and aminoglycosides, and increased sensitivity to negatively charged carbenicillin and chlortetracycline. Another gene of *L. lactis* MUT78.2 mutated in response to BHT-B encodes YnhD, a hypothetical protein nearly identical with lactococcal XpaC-like and 5-bromo-4-chloroindolyl phosphate hydrolysis proteins. Since its mutation was accompanied by SNPs in *ysaB* and *dxsA* genes, its direct role in resistance to tested antimicrobials remains uncertain, however, based on some previous reports we can anticipate that it also protects cells from some of the antimicrobials tested in this study. For example, the *ynhD* gene was found to be upregulated in the nisin resistant *L. lactis* cells where YnhD was annotated as a tellurite resistance protein [32]. Overexpression of tellurite resistance-related proteins (YceGHI) contributed to nisin resistance of *B. subtilis* [60]. Moreover, a gene encoding the XpaC-like protein was upregulated in the hBD3-treated *S. aureus* cells [61] and amino acid substitution within XpaC was found in daptomycin-resistant *E. faecium* [62]. However, further studies on the *ynhD* gene including its deletion and overexpression are required to evaluate its direct role in the resistance to AurA53- and EntL50-like bacteriocins and its interconnection with increased resistance or sensitivity to certain antibiotics.

Altogether, this is a first report on the genetic basis of resistance to AurA53- and EntL50-like bacteriocins in the interconnection with antibiotics. Bacteria may probably cope with these stressors by the accumulation of SNPs resulting in modification of the activity of certain enzymes that are, in the physiological state of cells, involved in lipid metabolism. The activity of such modified lipid metabolism DgkB and DxsA enzymes may lead to alterations in the composition of the cell envelope that lower the negative charge of the cell surface or increase the stability and integrity of the cytoplasmic membrane. The second-line protection, which further increases the cell resistance, includes the modification of the Bce-like stress response regulatory system proteins such as an ABC transporter permease YsaB. These changes enhance cell resistance primarily against lipid II cycle and membrane-acting antimicrobials, whereas having no major effect on intracellular target antibiotics. Our additional finding that decreased, via SNPs accumulation, sensitivity to some antimicrobials (membrane-active bacteriocins and antibiotics) results in the concurrently increased vulnerability to other ones (bacitracin, carbenicillin, chlortetracycline) is a premise of the design of multidrug preparations with tailored composition to reduce the risk of resistance development.

## 4. Materials and Methods

### 4.1. Bacterial Strains, Plasmids, and Culture Conditions

The bacterial strains and plasmids used in this study are listed in Table 3. Nisin-producing *L. lactis* IBB51 and Lcn972-producing *L. lactis* IPLA 972 were grown in M17 medium (BioMaxima, Lublin, Poland) supplemented with 0.5% (wt/vol) glucose (GM17) at 30 °C. Wild-type *L. lactis* LMGT 3419 and its derivates with random mutations or deletion of the *dxsA* gene were grown in Brain Heart Infusion (BHI) medium (Oxoid, Hampshire, UK) at 30 °C. *L. lactis* LMGT 3419- and *L. lactis* MUT78-derived mutants carrying a pIBB-JZK plasmid with the cellobiose-inducible promoter P*ptcB* were grown in GM17 medium at 30 °C. To induce transcription of the *dxsA* gene cloned under P*ptcB*, mutants were cultured in the M17 medium supplemented with 1% (wt/vol) cellobiose (CM17). *E. coli* TG1 and EC1000 were grown in Luria–Bertani (LB) medium (Becton, Dickinson and Company, East Rutherford, USA) at 37 °C. When appropriate, erythromycin (Ery) was added to a final concentration of 75 µg/mL for *E. coli* or 5 µg/mL for *L. lactis*, and ampicillin (Amp) or tetracycline (Tet) were added to a final concentration of 100 or 10 µg/mL, respectively. To prepare soft agar (soft BHI-agar) and agar plates (BHI-agar, GM17-agar, CM17-agar) liquid media were supplemented with agar (Merck, Darmstadt, Germany) to 0.75% and 1.5% (wt/vol), respectively.

### 4.2. Bacteriocin Preparation

BHT-B (MWGRILAFVAKYGTKAVQWAWKNKWFLLSLGEAVFDYIRSIWGG), K411 (MAGFLKVVKAVAKYGSKAVKWCWDNKGKILEWLNIGMAVDWIVEQVRKIVGA), Ent7 (MGAIAKLVAKFGWPIVKKYYKQIMQFIGEGWAINKIIDWIKKHI), EntL50 (MGAIAKLVAKFGWPIVKKYYKQIMQFIGEGWAINKIIEWIKKHI; MGAIAKLVTKFGWPLIKKFYKQIMQFIGQGWTIDQIEKWLKRH), WelM (MVSAAKVALKVGWGLVKKYYTKVMQFIGEGWSVDQIADWLKRH) and SalC (MSALAKLIAKFGYKKIMQLIGEGWTVNQIEKMFK) lyophilized bacteriocins with a purity of over 95% were synthesized by a commercial service (PepMic, Suzhou, China). Before use, the bacteriocins were dissolved in 0.1% trifluoroacetic acid (TFA) (Sigma, Darmstadt, Germany) to a final concentration of 1 mg/mL. Nisin and Lcn972 were precipitated from the 0.45 µm pore size filter-sterilized (MilliporeSigma, Burlington, MA, USA) supernatants of *L. lactis* IBB51 and *L. lactis* IPLA 972 overnight 100 mL cultures, respectively. The supernatants were saturated with 30% (wt/vol) ammonium sulfate (Sigma, Darmstadt, Germany) and stored for 1 h at 4 °C. The bacteriocin pellets were collected after centrifugation at 11,000 rpm for 40 min at 4 °C and dissolved in 1 mL of sterile water to the final concentration of 100×. The concentration of bacteriocins was estimated with Bradford protein assay on ND-1000 Spectrophotometer (NanoDrop Technologies, Inc., Wilmington, NC, USA).

### 4.3. Selection of BHT-B Resistant Mutants

Spontaneous resistant mutants were generated by growing *L. lactis* LMGT 3419 in the presence of BHT-B at a concentration of 0.01 mg/mL according to the method described before [41]. The level of sensitivity decrease was determined by growing mutants in the microtiter plates with serial two-fold bacteriocin dilutions as described before [41]. The minimum inhibitory concentration (MIC_50_) value was presented in μg/mL and defined as the lowest concentration of the bacteriocins, at which more than 50% of bacterial growth was inhibited.

### 4.4. DNA Isolation and Manipulation

Genomic DNA of the resistant mutants was isolated using Genomic Mini Kit (A&A Biotechnology, Gdynia, Poland) and prepared for sequencing with Nextera XT DNA Sample Preparation Kit, Nextera XT Indexing Kit and PhiX Control V3 Kit (Illumina, San Diego, CA, USA) according to the manufacturer’s instructions. Sequencing was performed using Miseq Sequencer (Illumina, San Diego, CA, USA) and the data were analyzed with CLC Genomics Workbench 8.5 (Qiagen, Hilden, Germany). Plasmid DNA was isolated using Plasmid Mini Kit or Plasmid Midi AX Kit (A&A Biotechnology, Gdynia, Poland). PCR reactions with the Phusion (Thermo Fisher Scientific, Waltham, MA, USA) or ExTaq (TaKaRa Bio Inc., Shiga, Japan) polymerases were carried out according to the manufacturer’s instructions. Plasmid DNA and PCR products were digested with FastDigest enzymes (Thermo Fisher Scientific) and ligated with T4 DNA ligase (Thermo Fisher Scientific, Waltham, MA, USA). DNA fragments from PCR, restriction digest, or agarose gel were purified using Wizard^®^ SV Gel and PCR Clean-Up System (Promega, Fitchburg, MA, USA). The translation of nucleotide sequences to protein sequences was done with an Expasy translate tool [67] (https://web.expasy.org/translate/). Protein sequences were aligned using the Clustal Omega program [68] (https://www.ebi.ac.uk/Tools/msa/clustalo/). Template-based tertiary structures of DxsA and YecE were modeled with the I-TASSER server [69] (https://zhanglab.ccmb.med.umich.edu/I-TASSER/). **1**-deoxy-D-xylulose **5**-phosphate synthases from *D. radiodurans* R1 and *E. coli* K12 (PDB/NCBI accession nos 2O1X/WP_010888114.1 and 2O1S/WP_074526961.1, respectively) and soluble diacylglicerol kinases from *S. aureus* MRSA252 and *E. faecalis* V583 (PDB accession nos 2QV7/WP_001231458.1 and 4WER/WP_002362274.1, respectively) were used as templates of the highest significance for DxsA and YecE, respectively. Predicted tertiary structures were visualized using PyMOL Molecular Graphics System, Version 2.0 (Schrödinger, LLC, New York, NY, USA).

### 4.5. Construction of the Deletion Mutant

Knockout of the *dxsA* gene was performed using the homologous recombination method. First, DNA fragments upstream (UP) and downstream (DN) of the *dxsA* gene were amplified with Phusion, and DxsAUPfor/rev and DxsADNfor/rev primers pairs (Table 3). Then, purified PCR products were digested with BamHI and ligated. An additional PCR with ExTaq and DxsAUPfor/DxsADNrev primers was performed to amplify ligated fragments. PCR product was purified from the agarose gel and cloned into pGEM-T Easy vector (Promega) by TA cloning. Ligated vector was transformed into *E. coli* TG1 and the sequence of the *dxsA* was verified by sequencing with 1224/1233 primers (Table 3). Next, the recombinant vector was digested with ApaI and NotI, the insert was purified from the agarose gel and cloned into pGhost9 vector. Ligated vector was transformed sequentially into *E. coli* EC1000 and *L. lactis* LMGT 3419. The presence of the insert was confirmed by PCR with ExTaq and pGhfor/rev primers (Table 3). Double cross-over event was forced by 10^3^-fold dilution of the *L. lactis* overnight culture in BHI medium supplemented Ery (5 µg/mL) and incubation for 1.5 h at 30 °C and 3.5 h at 37 °C. To select integrants, culture was streaked on BHI-agar plates supplemented Ery (5 µg/mL) and incubated at 37 °C. To remove the integrated vector, single colonies were passaged on BHI-agar plates and incubated at 30 °C. The deletion of the *dxsA* gene was confirmed by colony PCR with ExTaq and DxsAOUTfor/rev primers (Table 3). To cure the vector, the deletion mutant was cultivated in the absence of antibiotics at 30 °C. The absence of the pGhost9 was confirmed by colony PCR with ExTaq and pGhfor/rev primers, and tests of susceptibility to Ery.

### 4.6. Construction of the Expression Mutants

Expression of the dxsA gene was performed using the low-copy pIBB-JZK with cellobiose-inducible promoter PptcB [70]. First, dxsA was amplified using Phusion and DxsAfor/rev primers (Table 3). PCR product was purified and cloned into pIBB-JZK under PptcB using the BamHI and XhoI. Then, the ligated vector was transformed into *E. coli* EC1000 and the sequence of the dxsA was verified by sequencing with pIBB-JZKfor/rev primers (Table 3). Finally, the recombinant vector (pIBB-JZK:PptcB::dxsA) was transformed into *L. lactis* LMGT 3419 and *L. lactis* MUT78 resulting in the *L. lactis* MUT83 and *L. lactis* MUT85, respectively. The presence of the insert was confirmed by PCR with ExTaq and pIBB-JZKfor/rev primers.

### 4.7. Antibiotic Susceptibility Testing

Wild-type *L. lactis* LMGT 3419 and its derivates with random mutations or *dxsA*-deletion were streaked onto BHI-agar plates while *L. lactis* LMGT 3419- and *L. lactis* MUT78-derivates with pIBB-JZK were streaked onto CM17-agar plates supplemented Tet (10 µg/mL). Plates were incubated overnight at 30 °C. Suspensions of the strains were prepared in 0.85% saline and the turbidity was adjusted to that of McFarland 1.0 standard (bioMérieux, Marcy-l’Étoile, France). Then, the solutions were spread evenly over the surface of ISO-BHI-agar plates (Oxoid, Hampshire, UK) or CM17-agar plates supplemented Tet (10 µg/mL) and disks or strips impregnated with antibiotics were applied onto the plates. All antibiotic strips were purchased from bioMérieux except bacitracin strips that were purchased from Liofilchem (Roseto Degli Abruzzi, Italy). Carbenicillin and chlortetracycline disks were purchased from Oxoid and Čaderský-Envitek (Brno, Czech Republic), respectively. The plates were incubated for 48 h at 30 °C and then the inhibition zones were measured (mm). The MIC value (µg/mL) was defined as the concentration of the antibiotic at which the edge of the inhibition ellipse intersects the strip. Resistance to gramicidin (Sigma, Darmstadt, Germany) was determined using microtiter plates with serial two-fold gramicidin dilutions as described before [41].

## Figures and Tables

**Table 1:** Features of spontaneous *Lactococcus lactis* LMGT 3419 mutants resistant to BHT-B.

Mutant	Sensitivity Decrease *	Mutation in Nucleotide Sequence	Mutation in Amino Acid Sequence	Potential Function
MUT70	4×	286G→T in *dgkB* (*yecE*)	Asp96→Tyr	Diacylglycerol kinase
MUT72	4×	163G→T in *dgkB* (*yecE*)	Glu55→STOP	Diacylglycerol kinase
MUT71, MUT73, MUT78	4×	1469C→G in *dxsA*	Ser490→STOP	**1**-deoxy-D-xylulose **5**-phosphate (DXP) synthase
MUT78.2	8×	1469C→G in *dxsA*; 780T→G in *ysaB*; 328G→T in *ynhD*	Ser490→STOP;Tyr260→STOP; Glu110→STOP	DXP synthase; ABC transporter permease; hypothetical protein

* In comparison to the wild-type strain *L. lactis* LMGT 3419.

**Table 2:** Level of resistance of *L. lactis* LMGT 3419 wild-type and its mutants to aureocin A53-like and enterocin L50-like bacteriocins, nisin, lactococcin 972, and antibiotics. Colored resistance scale represents rounded fold-change of MIC (minimum inhibitory concentration) value relative to wild-type *L. lactis* LMGT 3419. Significantly increased or decreased diameters of bacterial growth inhibition are highlighted with vivid green or red, respectively.

Antimicrobial Agent	LMGT 3419	MUT70			MUT72		MUT78	MUT78.2	MUT80	MUT84	MUT85
	Wild-Type	*dgkB*^-^ (*yecE*-)			*dgkB*^-^ (*yecE*-)		*dxsA* ^-^	*dxsA* ^-^ *ysaB* ^-^ *ynhD* ^-^	∆*dxsA*	*dxsA*^-^ pIBB-JZK	*dxsA*^-^ pIBB-JZK:*dxsA*
	MIC_50_ [µg/mL]										
K411	3.2	6.4			6.4		12.5	>25	3.2	12.5	6.3
BHT-B	6.3	25			25		25	>25	6.3	25	12.5
Ent7	0.2	0.4			0.4		1.6	6.4	0.2	0.4	0.2
EntL50	0.2	0.8			0.8		6.4	25	0.4	0.8	0.8
WelM	2.4	18.8			18.8		18.8	>25	2.4	>25	25
SalC	6.3	12.5			12.5		>25	>25	6.3	25	12.5
Nisin	0.47	1.9			1.9		>15	>15	0.47	>15	15
Lcn972	0.4	1.6			1.6		3.2	>3.2	0.4	3.2	1.6
	MIC [µg/mL]										
Bacitracin	12	4			3		4–6	2	12	6	6
Daptomycin	0.094	1			0.5		1	2	0.064	0.5	0.5
Gramicidin	0.023	0.188			0.188		>0.75	0.75	0.012	0.75	0.75
Gentamicin	2	12			8–12		8	12–16	2	6	6
Kanamycin	12	32			32		32	32	6	32	32
Streptomycin	24	48			48		48	64	12	64	64
Vancomycin	0.38	1			0.5–0.75		1	1.5	0.5	1	1
Fosfomycin	384	>1024			>1024		1024	>1024	>1024	384	384
	Diameter [mm]										
Carbenicillin	28	31			31		32	33–34	29	33	34
Chlortetracycline	21	29			32		33	32	24	14 *	15 *
Sensitivity scale:	6×	4×		3×		2×	2×	4×	8×	16×	≥32×
Fold of increased sensitivity**←**							**→** Fold of decreased sensitivity				

*L. lactis* LMGT 3419 mutants did not exhibit significant changes in the sensitivity to antibiotics such as inhibitors of cell wall biosynthesis (amoxicillin, ampicillin, cefuroxime, and cephalothin), inhibitors of DNA biosynthesis (ciprofloxacin and norfloxacin), inhibitors of protein biosynthesis (chloramphenicol, clindamycin, and erythromycin), inhibitor of folate biosynthesis (trimethoprim), inhibitor of DNA, RNA and protein biosynthesis, respiration, and pyruvate metabolism (nitrofurantoin). * The significant decrease of sensitivity observed here is due to the *tetR* resistance gene present in pIBB-JZK.

**Table 3:** Bacterial strains, plasmids, and primers used in this study.

Strains, Plasmids, Primers	Description, Primer Sequence	Source * (Reference)
Strains		
*Lactococcus lactis*		
IBB51	Nisin producer	IBB PAS
IPLA 972	Lcn972 producer	IPLA-CSIC [63]
LMGT 3419	Indicator strain	LMGT
MUT70, MUT71, MUT72, MUT73, MUT78	LMGT 3419 spontaneous first generation mutants resistant to AurA53- and EntL50-like bacteriocins	This study
MUT78.2	LMGT 3419 spontaneous second-generation mutant resistant to AurA53- and EntL50-like bacteriocins	This study
MUT79c	LMGT 3419 strain carrying pGhost9:Δ*dxsA*	This study
MUT80	LMGT 3419 strain with *dxsA* deletion	This study
MUT82	LMGT 3419 strain carrying pIBB-JZK	This study
MUT83	LMGT 3419 strain carrying pIBB-JZK:*dxsA*	This study
MUT84	MUT78 strain carrying pIBB-JZK	This study
MUT85	MUT78 strain carrying pIBB-JZK:*dxsA*	This study
*Escherichia coli*		
TG1	Host strain, Δ(*hsdMS-mcrB*) 5Δ(*lac-proAB*) *supE thi-1* F’(*traD36 proAB*^+^*lacI*^q^*Z*Δ*M15*)	[64]
EC1000	Host strain, Kmr, *repA+* derivative of MC1000, carrying a single copy of the pWV01 *repA* gene in *glgB*	[65]
Plasmids		
pGEMT	Amp^r^, M13*ori*, linear T-overhang vector	Promega
pGhost9	Em^r^, *repA*(Ts)	IBB PAS [66]
pIBB-JZK pIBB-JZK:P*ptcB*::*dxsA*	Amp^r^,Tet^r^, cellobiose-responsive promoter (P*ptcB*) Amp^r^,Tet^r^, *dxsA* cloned under the control of P*ptcB*	IBB PAS
Primers	DNA sequence (5′->3′), restriction site **	
1224/1233	CGCCAGGGTTTTCCCAGTCACGA/AGCGGATAACAATTTCACACAGG	
pGhfor/rev	TGTAAAACGACGGCCAGTG/AGTACCGTTACTTATGAGC	
pJZK-IBBfor/rev	AGTCGCCTAAAGGTTGC/CGATGTTCTGTCCCTTG	
DxsAUPfor/rev	CAAGTATGCTCCAAGG/GAGGATCCCGAGCTCTTCTAGTTC	
DxsADNfor/rev	GAGGATCCGCAGAGCTTATCTCCTA/CCAGCAGAATGGAAAC	
DxsAOUTfor/rev	GAGGAGTCCTCCAAATG/TCTGTAGATGTCACGG	
DxsAfor/rev	CGATGGATCCCTAAATAGAACTAGGAAG/CGATCTCGAGGAGATAAGCTCTGCCTAC	

* Bacterial strains derived from the Regional Strains and Plasmids Collection of the Institute of Biochemistry and Biophysics, Polish Academy of Sciences, Warsaw, Poland (IBB PAS); from the collection of the Laboratory of Microbial Gene Technology, Department of Chemistry, Biotechnology and Food Science, Norwegian University of Life Sciences, Ås, Norway (LMGT); from the collection of the Dairy Institute of Asturias, Spanish National Research Council, Asturias, Spain (IPLA-CSIC) and were obtained in this study. ** BamHI and XhoI restriction sites are underlined.

## Data Availability

In this section, please provide details regarding where data support.
